# Lessons From Prospective Longitudinal Follow-up of a French APECED Cohort

**DOI:** 10.1210/clinem/dgae211

**Published:** 2024-04-12

**Authors:** Linda Humbert, Emmanuelle Proust-Lemoine, Sylvain Dubucquoi, Elisabeth Helen Kemp, Pascale Saugier-Veber, Nicole Fabien, Isabelle Raymond-Top, Catherine Cardot-Bauters, Jean-Claude Carel, Maryse Cartigny, Olivier Chabre, Philippe Chanson, Brigitte Delemer, Christine Do Cao, Laurence Guignat, Jean Emmanuel Kahn, Veronique Kerlan, Herve Lefebvre, Agnès Linglart, Roberto Mallone, Rachel Reynaud, Boualem Sendid, Pierre-François Souchon, Philippe Touraine, Jean-Louis Wémeau, Marie-Christine Vantyghem

**Affiliations:** Department of Endocrinology, Diabetology and Metabolism, Huriez Hospital, Lille University Hospital, F-59000 Lille, France; Department of Endocrinology, Diabetology and Metabolism, Huriez Hospital, Lille University Hospital, F-59000 Lille, France; Institut d’Immunologie-HLA, Centre de Biologie-Pathologie, 59037 Lille Cedex, France; University of Lille, 59000 Lille, France; Department of Oncology and Metabolism, Faculty of Medicine, Dentistry and Health, University of Sheffield, Medical School, Sheffield S10 2RX, UK; Department of Genetics and Reference Center for Developmental Disorders, Univ Rouen Normandie, Inserm U1245, Normandie Univ and CHU Rouen, F-76000 Rouen, France; Laboratory of biology, CHU Lyon, 69 000 Lyon Cedex, France; Institut d’Immunologie-HLA, Centre de Biologie-Pathologie, 59037 Lille Cedex, France; Department of Endocrinology, Diabetology and Metabolism, Huriez Hospital, Lille University Hospital, F-59000 Lille, France; Service d’Endocrinologie Diabétologie Pédiatrique and INSERM NeuroDiderot, Centre de Référence Maladies Endocriniennes Rares de la Croissance, AP-HP Nord Université Paris Cité, Hôpital Universitaire Robert-Debré, 75935 Paris Cedex 19, France; Department of Pediatry, Hôpital Jeanne de Flandres, Lille University Hospital, F-59000 Lille, France; Unité mixte de recherche INSERM-CEA-UGA UMR1036, Service d’Endocrinologie CHU Grenoble Alpes, Université Grenoble Alpes, 38000 Grenoble Alpes, France; Inserm, Physiologie et Physiopathologie Endocriniennes, Assistance Publique-Hôpitaux de Paris, Hôpital Bicêtre, Service d’Endocrinologie et des Maladies de la Reproduction, Centre de Référence des Maladies Rares de l’Hypophyse, Université Paris-Saclay, 94275 Le Kremlin-Bicêtre, France; Department of Endocrinology and Diabetology, CHU Reims, 51 092 Reims, France; Department of Endocrinology, Diabetology and Metabolism, Huriez Hospital, Lille University Hospital, F-59000 Lille, France; Centre de Référence des Maladies Rares de la Surrénale, Endocrinologie, Hôpital Cochin, 75014 Paris, France; Institut d’Immunologie-HLA, Centre de Biologie-Pathologie, 59037 Lille Cedex, France; Department of Internal Medicine, National Reference Center for Hypereosinophilic Syndromes (CEREO), Hôpital Foch, 92151 Suresnes, France; APHP, CHU Ambroise Paré, University of Paris Saclay, 92104 Boulogne-Billancourt, France; Department of Endocrinology, Diabetology and Metabolism CHU Brest, Hôpital de la Cavale Blanche, 29609 Brest Cedex, France; Department of Endocrinology, University Hospital of Rouen, 76031 Rouen, France; AP-HP, Service d'Endocrinologie et Diabète de l'Enfant, Hôpital Bicêtre Paris-Saclay, AP-HP, Centre de Référence des Maladies Rares du Métabolisme du Calcium et du Phosphate, Filière OSCAR, ERN BOND, ERN for Rare Endocrine Disorders, Plateforme d'Expertise des Maladies Rares de Paris Saclay, INSERM U1185, Université Paris Saclay, 94270 Le Kremlin-Bicêtre, France; Clinical Department of Diabetology and Clinical Immunology, INSERM U1016 Cochin Institute, DeARLab Team Mallone-You, Groupe Hospitalier Cochin-Port-Royal, 75014 Paris, France; Service de Pediatrie Multidisciplinaire, CHU Timone Enfants, Centre de Reference Maladies Hypophysaire Rares, APHM Aix Marseile Université 13385, Marseille Cedex 05, France; Institut de Microbiologie, Centre de Biologie Pathologie Génétique, Inserm U1285—CNRS UMR 8576, Centre Hospitalier Universitaire de Lille, 59037 Lille, France; CHU de Reims—American Memorial Hospital—Pediatric Department, 51092 Reims Cedex, France; Department of Endocrinology and Reproductive Medicine, AP-HP, Sorbonne University Medicine, 75013 Paris, France; Department of Endocrinology, Diabetology and Metabolism, Huriez Hospital, Lille University Hospital, F-59000 Lille, France; University of Lille, 59000 Lille, France; Department of Endocrinology, Diabetology and Metabolism, Huriez Hospital, Lille University Hospital, F-59000 Lille, France; University of Lille, 59000 Lille, France; Inserm U1190, European Genomic Institute for Diabetes, Lille University, F-59000 Lille, France

**Keywords:** APECED syndrome, autoimmune polyendocrine syndrome type 1, *AIRE* genotype, asplenia, pulmonary involvement

## Abstract

**Background:**

Autoimmune polyendocrinopathy-candidiasis-ectodermal dystrophy syndrome is a rare disease caused by biallelic mutations of the *AIRE* gene, usually presenting with the triad hypoparathyroidism-adrenal failure-chronic mucocutaneous candidiasis (CMC) and nonendocrine manifestations. The aim of this study was to determine the molecular profile of the *AIRE* gene, the prevalence of rare manifestations, and to characterize immunological disturbances in a French cohort.

**Patients and Methods:**

A national, multicenter prospective observational study to collect genetic, clinical, biological, and immunological data (NCT03751683).

**Results:**

Twenty-five patients (23 families) were enrolled. Eleven distinct *AIRE* variants were identified, 2 of which were not previously reported: an intronic variant, c.653-70G > A, and a *c.1066del (p.Arg356GlyfsX22)* variant (exon 9). The most common was the Finnish variant *c.769C > T* (16 alleles), followed by the variant c.967_979del13 (15 alleles), which seemed associated with a less severe phenotype. Seventeen out of 25 patients were homozygote. The median number of clinical manifestations was 7; 19/25 patients presented with the hypoparathyroidism-adrenal failure-CMC triad, 8/13 showed pulmonary involvement, 20/25 had ectodermal dystrophy, 8/25 had malabsorption, and 6/23 had asplenia. Fifteen out of 19 patients had natural killer cell lymphopenia with an increase in CD4^+^ and CD8^+^ T lymphocytes and an age-dependent alteration of B lymphocyte homeostasis compared with matched controls (*P* < .001), related to the severity of the disease. All tested sera (n = 18) were positive for anti-interferon-α, 15/18 for anti-IL-22 antibodies, and 13/18 for anti-IL-17F antibodies, without clear phenotypic correlation other than with CMC.

**Conclusion:**

This first prospective cohort showed a high *AIRE* genotype variability, with 2 new gene variants. The prevalence of potentially life-threatening nonendocrine manifestations was higher with systematic screening. These manifestations could, along with age-dependent B-cell lymphopenia, contribute to disease severity. Systematic screening for all the manifestations of the syndrome would allow earlier diagnosis, supporting vaccination and targeted therapeutic approaches.

Autoimmune polyendocrine syndrome type 1 (APS-1; OMIM 240300), also known as autoimmune polyendocrinopathy-candidiasis-ectodermal dystrophy (APECED), is characterized by the clinical triad of hypoparathyroidism, adrenal insufficiency, and chronic mucocutaneous candidiasis (CMC). This syndrome was formerly known as Whitaker syndrome (1). Accurate diagnosis of the syndrome requires the presence of at least 2 of these 3 major components or only 1 if a sibling has already been diagnosed with the disease (2). APECED can be associated with other autoimmune endocrine disorders, such as autoimmune ovarian or testicular failure, thyroid disease, type 1 diabetes, and hypophysitis, or with nonendocrine autoimmune disease, eg, celiac disease, hepatitis, alopecia, vitiligo, keratitis, and chronic atrophic gastritis. These autoimmune disorders are associated with ectodermal dystrophy, asplenia, and the presence of several autoantibodies, even in the absence of corresponding organ dysfunction (3). APS-1 is usually a monogenic, autosomal recessive disease caused by variants of the autoimmune regulator (*AIRE*) gene on chromosome 21 (MIM#607358) (4). The *AIRE* gene codes for a nuclear transcriptional regulator protein involved in the ectopic expression of self-antigens mainly in the thymus, leading to the removal of self-reactive thymocytes and the generation of central tolerance. Different mechanisms have been investigated such as impaired production of thymic regulatory T cells through modification of CTLA-4 expression from the thymic stroma (5) and increase of CD45RC in peripheral blood T cells and lesioned organs (6). To date, more than 100 different mutations in the *AIRE* gene, both homozygous and heterozygous, have been reported (7-9). APECED is a rare syndrome, which has been reported worldwide but is more prevalent in some historically isolated homogeneous populations, especially in Finland (1/25 000) (4, 10), in Sardinia (1/14 500), (11) and among Iranian Jews (1/9000) (12). APECED also has a lower incidence in Norway, Sweden, Slovenia, Great Britain, Italy, Ireland, and North America (13-18).

In France, a first retrospective study was performed in the northwest region (19). A larger study was needed to complete the collected data, and a national Hospital Clinical Research Program (NCT03751683) was launched in 2009 to prospectively characterize the phenotype and genotype of a national cohort. The main objectives in this genetically confirmed French APECED cohort were to define the molecular profile to determine the total number of patients who could be enrolled regardless of the diagnostic criteria used, to describe the clinical components, and to report the immunological characteristics of the disease. Our aims were (1) to establish a link between variants of the *AIRE* gene and the occurrence of clinical manifestations, (2) to detect potentially serious but also milder forms, and (3) to assess the clinical course of the disease with a 2-year follow-up to optimize the recognition and treatment of these patients.

## Patients and Methods

### Study Design

Recruitment for this clinical research study was first announced at French national endocrine meetings. Patients with already known or clinically suspected APECED were offered to participate in this prospective national study if they agreed to be seen in an authorized investigation center.

### Patients

The inclusion criteria were APS-1 already confirmed on a molecular basis or patients presenting with classical diagnostic criteria (at least 2 components of the Whitaker's triad or only 1 component in siblings of APECED patients) (20), regardless of age and sex. To detect underdiagnosed or poorly symptomatic forms of the disease, we added another inclusion criteria, which was a genetic diagnosis in case of 1 major component associated with 2 minor components, including peripheral hypogonadism, atrophic gastritis, intestinal malabsorption, autoimmune hepatitis, vitiligo, alopecia, keratoconjunctivitis, and dental enamel hypoplasia. Thyroiditis and type 1 diabetes mellitus were not included in this list of minor components because they are very frequent; nevertheless, they were not exclusion criteria.

### Outcomes

Enrolled patients were prospectively followed for 3 yearly visits. Patients who had not been previously tested for *AIRE* variants were tested at the screening visit (V0). In the absence of *AIRE* mutation, patients were excluded. At visit 0, the personal and familial medical history was recorded. At visits 0, 1 year, and 2 years, a complete clinical examination including endocrine, dermal, oral, ophthalmic, pulmonary, and gastroenteric components was performed. Patients underwent a lab screening including routine, hormonal, and immunologic data (autoantibody screening and lymphocyte phenotyping; see later discussion). Abdominal ultrasonography and lung function tests were performed at the 3 visits.

This study was approved by the hospital ethics committee and was registered as project no. 1927 “Genotypic, Phenotypic and Prognostic Evaluation of APECED Syndrome: A National Multicenter Study.” Written informed consent was obtained from all patients or from both parents for patients under 18 years prior to visit 0, both for the genetic study and study participation.

### Methods

#### *AIRE* gene variants analysis

*AIRE* gene variants were analyzed in 2 steps by Sanger sequencing. The first step involved sequencing of exons 6 and 8, and then, if no variant or only 1 variant was found, the other 12 exons and exon-intron boundaries of the *AIRE* gene were analyzed by PCR as previously described (21). The variants were reported according to standardized nomenclature defined by the reference human genome GRCh37/hg19 and the *AIRE* isoform NM_0003834.

#### Autoantibody analysis

Antibodies were detected by routine methods in the local laboratory of each hospital and included those against 21-hydroxylase (AB_3094515), smooth muscle, liver-kidney microsomal antigen (AB_3094522), parietal gastric cells (AB_3094520), intrinsic factor (AB_3094521), islet cells (ICA) (AB_3094518), glutamic acid decarboxylase 65 (AB_3094514), tyrosine phosphatase-like protein (IA-2) (AB_2910240), thyroperoxidase (AB_3094511), and thyroglobulin (AB_3094512).

Serum anti-parathyroid autoantibodies were measured by indirect immunofluorescence against human parathyroid tissue, and antibodies against the parathyroid calcium-sensing receptor were detected using an immunoblotting assay with the recombinant extracellular domain of the calcium-sensing receptor (CaSR), as previously described (22). Antibodies against CaSR (AB_3094526), NACHT leucine-rich-repeat protein 5 antibodies (NALP5) (AB_3094524), and cytokines (AB_30_94525) were measured only at the first visit using immunoprecipitation, radioligand binding, and ELISA assays, respectively, as previously reported (23).

#### Lymphocyte immunophenotyping

Peripheral blood lymphocyte immunophenotyping by flow cytometry was performed at the first visit. Relative numbers of CD4/CD8+ T cells, B cells, and natural killer (NK) cells were determined using routine flow cytometry techniques (Navios Flow Cytometer, Beckman Coulter). In addition, flow cytometric analysis of naive and memory B cells was performed. To do so, whole blood samples underwent red cell lysis using Uti-Lyse (Dako) and were washed twice before staining. Absolute and relative numbers of naive, memory, and marginal zone-like subsets comprising the CD19+ B cell populations were determined by 4-color staining with antibodies to anti-CD19-PE-Cy7, in combination with anti-CD27-PE-Cy5 (both from Beckman Coulter), anti-IgD-PE, anti-IgM-FITC (both from Dako), and transitional subsets using anti-CD19-PE-Cy5 (Beckman Coulter), anti-IgM-FITC (Dako), and anti-CD38-PE (BD Biosciences). Naive and memory B cell subsets were expressed as a percentage of the total CD19+ B cells. Their absolute counts were calculated by multiplying the specific subset percentage by the absolute CD19+ B lymphocyte count as determined by Flow-Count fluorospheres.

Lymphocyte subpopulations and immunophenotype results were compared with a control group of healthy subjects corresponding to laboratory standards of the Immunology Department of the Center for Biology Pathology at Lille University Hospital. To overcome the effect of age on B lymphocyte homeostasis, APECED patients and controls were separated into 2 groups (<30 years old and >30 years old).

### Statistics

Statistics were performed by the Lille University Biostatistics Unit with SAS software, version 9.4. Fisher's exact test, chi-squared test, and Mann–Whitney U test were performed for analyses of the immunophenotypic characterization of peripheral blood lymphocytes where appropriate. A *P*-value less than .05 was considered statistically significant.

## Results

### Patient Demographics

**Figure 1.**
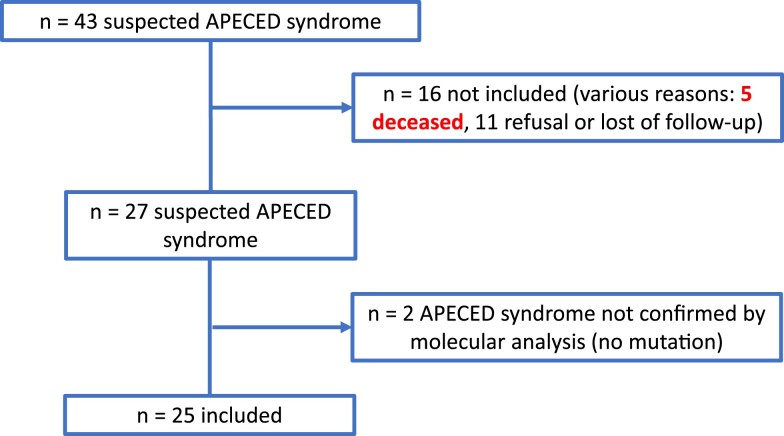
Study flow chart.

We identified 43 patients with suspected APECED, but only 25 patients could be included in the study for various reasons (Fig. 1). Twenty-five consecutive patients from 23 families were eventually enrolled over 7 years, including 8 from consanguineous families and 2 siblings. Thirteen patients (52%) were male and 12 (48%) were female. The sex ratio was 1.1, with a mean age of 26 years (range 5-52 years). Forty percent of patients were under 18 years of age. Almost 70% of patients were of European descent; the remaining 30% were of North African (n = 4), Senegalese (n = 2), Roma (n = 1), or French West Indies (n = 1) origin. No included patient died during the study period.

### Molecular Profile of the *AIRE* Gene

**Figure 2.**
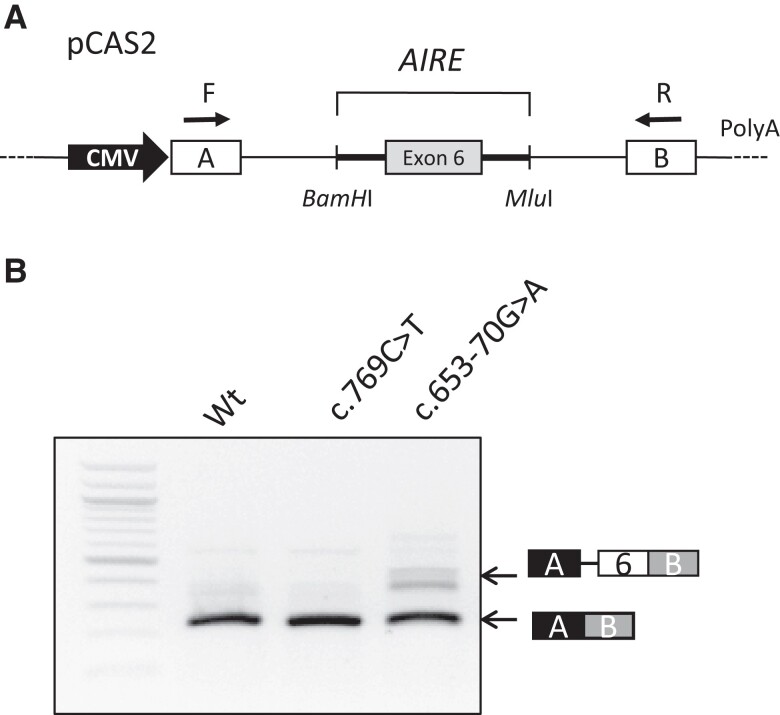
Effect on splicing of the c.653-70G > A variant located in intron 5 of the *AIRE* gene, assessed using a functional minigene assay. (A) Schematic representation of the pCAS2-AIRE minigenes used in the splicing reporter assay. Boxes indicate exons, whereas lines in between represent introns. The minigenes were generated by inserting a genomic fragment, containing exon 6 of the *AIRE* exon (gray box), as well as part of the upstream and downstream flanking intronic sequences (thick lines), into the intron of the minigene using the *BamH*I and *Mlu*I restriction sites. Expression of the minigenes is driven by the human cytomegalovirus promoter. Arrows above the minigene exons A and B (white boxes) indicate the positions of primers used in RT-PCR analysis. (B) Analysis of the splicing pattern of the Wt and mutant pCAS2-AIRE minigenes for the c.653-70G > A and c.769C > T variants. Wt and mutant pCAS-AIRE constructs were transiently expressed in HeLa cells by transfection. The splicing patterns of the minigene transcripts were then analyzed by RT-PCR as previously described (24).The image shows the electrophoresis on a 2% agarose ethidium bromide-stained gel of the RT-PCR products obtained for each minigene. The identities of the RT-PCR products are indicated on the right. The c.653-70G > A variant allows the retention of 68 bp of intron 5 in the final transcript and a frameshift as the c.769C > T variant, as expected, has no splice effect. Abbreviation: Wt, wild-type.

Biallelic variants were identified in all patients. The types and frequency of each variant are given in Table 1. The most frequent *AIRE* gene mutation, *c.769C > T* (exon 6), was present on 16 (35%) alleles and found in a homozygous state in 6 patients. Another *AIRE* gene variant, *c.967_979del13* (exon 8), was found in 15 (33.3%) alleles and in a homozygous state in 5 patients. Three patients showed the *c.1193delC* variant (exon 10) in the homozygous state. Some rare variants were also identified, including the *c.173C > A* variant in the homozygous state in 1 patient and the *c.274C > T* variant on 1 allele, both on exon 2; the *c.977C > T* variant and the *c.958del* variant, both on 1 allele in exon 8; the *c.1066del* variant (exon 9), the *c.1616C > T* variant (exon14), the *c.415C > T* variant (exon 3), each on 1 allele; and finally the *c.653-70G > A* variant in intron 5, which had never been described. To prove the pathogenicity of the intronic *c.653-70G > A* variant, we performed an ex vivo splicing test showing a splice defect by using a cryptic splice site, which resulted in the inclusion of 68 base pairs of intron 5 in the final transcript of the *AIRE* gene. This anomaly resulted in an interruption of the reading frame, thus confirming the deleterious nature of this variant (Fig. 2) (24).

**Table 1.** *AIRE* gene variants and clinical phenotypes

Variant nucleotidic (proteic); exon or intron	Clinical manifestations (number of patients)
c.769C > T *(R257X);* Exon 6: 16 alleles (6 homozygous), 10 patients	Hypoparathyroidism (10), CMC (9), adrenal insufficiency (9) Other endocrine manifestations: 5 patients Nonendocrine manifestations: 6 patients
c.967_979del13 (p.Leu323fs); Exon 8: 15 alleles (5 homozygous), 10 patients	Hypoparathyroidism (7), CMC (9), adrenal insufficiency (8), Other endocrine manifestations: 5 patients Nonendocrine manifestations: 9 patients
c.1193delC (p.Pro398fs); Exon 10: 6 alleles (3 homozygous), 3 patients	Hypoparathyroidism (3), CMC (3), adrenal insufficiency (3), Other endocrine manifestations: 2 patients Nonendocrine manifestations: 3 patients
c.958del (p.Leu320fs); Exon 8: 4 alleles (2 homozygous state), 2 patients	Hypoparathyroidism (2), CMC (2), adrenal insufficiency (2), Other endocrine manifestations: 1 patient Nonendocrine manifestations: 2 patients
c.173C > A (p.Ala58Asp); Exon 2: 2 alleles (1 homozygous), 1 patient	Hypoparathyroidism (1) CMC (1), adrenal insufficiency (1) Other endocrine manifestations: 0 patient, Nonendocrine manifestations: 1 patient
c.415C > T (p.Arg139Ter); Exon 3: 2 alleles, 2 patients	Hypoparathyroïdism (2), CMC (1), adrenal insufficiency (1) Other endocrine manifestations: 0 patient, Nonendocrine manifestations: 1 patient
c.977C > T (p.P326L); Exon 8: 1 allele, 1 patient	Hypoparathyroidism, adrenal insufficiency, CMC Other endocrine manifestations: 0 Nonendocrine manifestations: 2 manifestations
c.1066del (*p.Arg356GlyfsX22*); Exon 9: 1 allele, 1 patient	Hypoparathyroidism, CMC Other endocrine manifestations: 1 manifestation Nonendocrine manifestations: 2 manifestations
c.274C > T (p.Arg92Trp); Exon 2: 1 allele, 1 patient	Hypoparathyroidism, CMC, adrenal insufficiency Other endocrine manifestations: 3 manifestations Nonendocrine manifestations: 5 manifestations
c.1616C > T (p.Pro539Leu); Exon 14: 1 allele, 1 patient	Hypoparathyroidism, CMC, adrenal insufficiency Other endocrine manifestations: 1 manifestation Nonendocrine manifestations: 3 manifestations
c.653-70G > A (Intron 5): 1 allele, 1 patient	Hypoparathyroidism
**Clinical phenotype at inclusion: Number of patients (%)**	
**Whitaker triad**	19 (76)
Primary hypoparathyroidism	22 (88)
Adrenal insufficiency	22 (88)
Chronic cutaneomucosis candidiasis	22 (88)
*Onychomycosis*	14
*Oral mycosis*	14
*Oesophagal mycosis*	11
*Oesophagal stenosis*	2
*Cutaneous candidiasis*	8
* Vaginal candidiasis*	4
*Trichophytosis*	1
**Other endocrine manifestations**	
Hypergonadotropic hypogonadism	11 (44)
Type 1 diabetes	2
Hypothyroidism	2
Hypopituitarism	2
Graves disease	1
Atrophic gastritis	7 (28)
Malabsorption	8 (32)
**Nonendocrine manifestations**	
Ectodermal dystrophy	22 (80)
Vitiligo	7
Ungueal dystrophy	12
Enamel dystrophy	11
Alopecia	10
Keratoconjonctivitis	8
Dry eye syndrome	3
Splenic hypotrophy	6
Impaired lung function tests	8/13
Tubulointerstitial nephritis	1
Devic syndrome	1

The clinical phenotypes are classified according to the Whitaker triad (hypoparathyroidism-adrenal failure-chronic mucocutaneous candidiasis), other endocrine and nonendocrine manifestations, and then given by type and frequency.

*AIRE* gene variations c.769C > T (E6) and c.967_979del13 (E8) are the 2 most common variants, homozygous in 10 out of 21 patients, All variants are associated the most frequently with the Whitaker triad (sometimes incomplete, probably in relationship with the age of diagnosis). Whatever the variant, all patients showed nonendocrine manifestations, emphasizing the importance to screen for them. c.173C > A (E2), c.415C > T (E3), and c.977C > T (E8) were not associated with other endocrine manifestations. c.653-70G > A (I5) was associated with hypoparathyroidism only but in a young patient.

Abbreviation: CMC, chronic mucocutaneous candidiasis.

### Clinical Phenotype Including Nonendocrine Symptoms

#### At inclusion visit

The median age at diagnosis was 12 years (range 1 month-36 years). The median age at the first manifestation was 6 years (range 1 month-18 years).

The median number of manifestations at inclusion was 7 (range 1-13). At that time, 19 patients (76%) had the Whitaker's triad, 5 had 2 components of the triad, and 1 had isolated hypoparathyroidism with positive APS-1 molecular diagnosis before inclusion.

**Figure 3.**
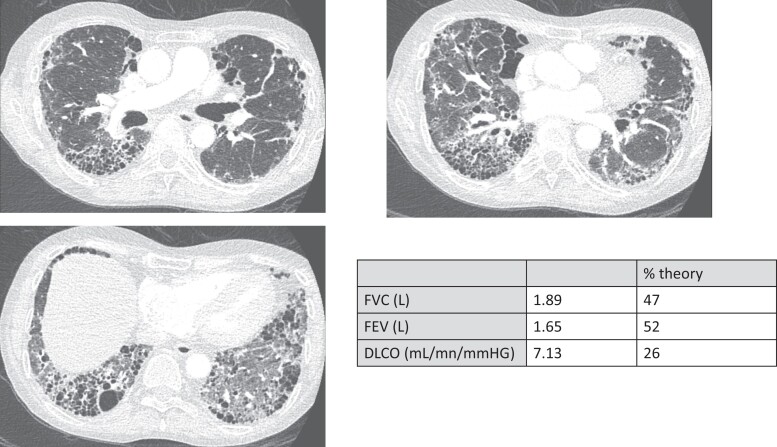
Patient #25 chest computed tomography scan and lung function tests. Bronchiectasis, honeycombing, micro and macrocystic parenchymal destruction, and ground-glass opacities. Abbreviations: FVC, forced vital capacity, FEV, forced expiratory volume in 1 second.

The first manifestation was CMC in 10 patients (40%), adrenal insufficiency in 7 (28%), hypoparathyroidism in 6 (24%), and atrophic gastritis associated with alopecia in 1 case. As shown in Table 1, at V0, 76% of the 25 patients had a Whitaker triad, 88% an adrenal insufficiency, 88% primary hypoparathyroidism, 44% a hypergonadotropic hypogonadism, 28% an atrophic gastritis, 32% a malabsorption, 92% a CMC, and 80% an ectodermal dystrophy; 6 patients had asplenia, 2 patients had a type 1 diabetes, 2 suffered a primary hypothyroidism, and 2 had a hypopituitarism. One patient had a Graves disease, 1 a tubulointerstitial nephritis, and 1 a Devic syndrome. At V0, 26% had asplenia. Concerning pulmonary symptoms at the inclusion visit, 7/25 patients were symptomatic and 18/25 had no pulmonary symptoms. At V0. 8 out of the 13 patients who completed lung function tests (LFTs) (62%) had impaired LFTs, among whom 4/8 were symptomatic. Of the patients who did not undergo LFTs, 3/12 were symptomatic, including recurrent pneumonitis (Fig. 3), asthma, and chronic cough. The cumulative frequencies of the main manifestations of APECED syndrome are detailed in Table 2. The frequency, sex ratio, and age at onset of the different manifestations are detailed in Table 3.

**Table 2.** Cumulative frequencies of the main manifestations of APECED syndrome

	0-10 years (%)	10-20 years (%)	20-40 years (%)	40-60 years (%)
Hypoparathyroidism	52	80	88	88
Adrenal insufficiency	36	76	88	88
Chronic mucocutaneous candidiasis	60	88	92	92
Hypogonadism	8	36	36	40
Atrophic gastritis	4	20	24	28
Malabsorption	4	24	24	24

Abbreviation: APECED, autoimmune polyendocrinopathy-candidiasis-ectodermal dystrophy.

**Table 3.** Frequency, sex ratio, and age at onset of the different manifestations

Manifestation	Frequency (%)	Sex-ratio (male/female)	Median age (min-max) (years)
Hypoparathyroidism	88	1	8.5 (2-27)
Adrenal insufficiency	88	1.4	11 (2-25)
Hypergonadotropic hypogonadism	44	0.1	14 (4-20)
Type 1 diabetes	8		24 (15-33)
Hypothyroidism	8		12 (9-15)
Chronic mucocutaneous candidiasis	92	1.1	11 (0.1-34)
Atrophic gastritis	28	0.75	16 (10-29)
Malabsorption	32	3	15 (7-47)
Autoimmune hepatitis	8		19.5 (3-36)
Vitiligo	28	0.75	12 (11-18)
Ungueal dystrophy	52	1.5	12 (5-18)
Enamel hypoplasia	48	0.83	12 (5-33)
Alopecia	40	1.5	12 (5-30)
Keratoconjunctivitis	33	0.6	24 (5-45)
Dry eye syndrome	12		23 (13-33)

#### Phenotype-genotype correlations

The phenotype and genotype associations are detailed in Tables 1 and  4.

**Table 4.** Phenotype and genotype of each patient

Family patient	Sex age (years) Consanguinity (bold)	Clinical manifestations (age of occurrence in year) in bold: total number of clinical manifestations/patient			Variant (E/exon or I/intron)
		Whitaker triad	Other endocrine	Nonendocrine	
A#1	F 5	CMC (1), HPT (5), **2**	0	0	c.415C > T (E3)/ c.967_979del13 (E8)
A#2	M 8	HPT (6), AI (8), **3**	0	B (UK)	c.415C > T (E3)/. c.967_979del13 (E8)
B#3	F 8	HPT (3), **1**	0	0	c.769C > T (E6)/ c.653-70G > A (I5)
D#6	F 13	CMC (3), HPT (7), **5**	HG (13)	EH (childhood), B (UK)	c.967_979del13(E8)/ c.1066del (E9)
F#8	M 14	CMC (4), HPT (6), AI (5), **3**	0	0	c.769C > T (E6)/ c.769C > T (E6)
G#9	M 15	CMC (6), AI (childhood), **5**	HP (childhood, GH deficiency)	UD (childhood),	c.967_979del3(E8)/ c.967_979del13 (E8)
I#11	M 18	CMC (13), HPT (13), AI (13), **5**	HG (16)	A (10),	c.769C > T (E6)/ c.769C > T (E6)
J#12	M 26	AI (12), CMC (16), **5**	0	VI (18), UD (18), A (18)	c.967_979del13 (E8)/ c.967_979del13 (E8)
N#16	M 31	CMC (8), HPT (10), AI (10), **4**	0	UD (UK)	c.967_979del13 (E8)/ c.967_979del13 (E8)
O#17	**M 32**	HPT (27), AI (25), CMC (childhood) **5**	0	AG (13), Mal (25)	c.1193delC(E10)/ c.1193delC (E10)
W#23	F 48	AI (18), HPT (22), CMC (34), **4**	HG (19)		c.769C > T (E6)/ c.769C > T (E6)
Y#25	M 52	HPT (10), AI (12), CMC (UK), **6**	0	KC (12), EH (33), B (UK)	c.769C > T(E6)/ c.967_979del13 (E8)
**< 7 manifestations**	**4 F/8 M** **Age range: 5-52 years Consanguinity: 1/12**	**n = 12 patients < 7 manifestations**	**3/12 HG** **1 HP/12**	**7/12**	10/24: 967_979del13(E8) 8/24: c.769C > T (E6) 2/24: c.1193delC (E10) 2/24: c.415C > T (E3) **7 homozygous**
C#4	**M 10**	CMC (3), HPT (3), AI (8), **8**	HG (4),	Mal. (7), UD (6), EH (6), TIN (UK), B (UK)	c.958del (E8)/c.958del (E8)
C#5	**F 13**	CMC (0,08), HPT (3), AI (7), **7**	HG (9), HT (9),	EH (childhood), KC (childhood)	c.1193delC(E10)/ c.1193delC (E10)
E#7	**M 13**	CMC (11), HPT (12), AI (12), **7**	0	EH (12), UD (12), Mal (13), AS (13)	c.958del (E8)/c.958del (E8)
H#10	M 16	CMC (0,08), AI (11), HPT (15)**, 10**	DT (15), HT (15),	V (12), EH (12), DES (13), AG (15), Mal (15), B (childhood)	c.769C > T(E6)/ c.1616C > T (E14)
K#13	**F 27**	HPT (2), AI (8), CMC (20), **12**	GD (UK), (18), HG (20),	HAI (3), AG (10), Mal (15), V (16), KC (UK), EH (25), AS (25), B(UK)	c.1193delC (E10)/ c.1193delC (E10)
L#14	**F 28**	AI (6), CMC (10), HPT (13), **7**	HG (13),	EH (13), UD (13), AG (17)	c.769C > T (E6)/ c.769C > T (E6)
M#15	M 28	CMC (11), HPT (11), AI (13), **9**	0	V (12), UD (12), A (12), AG (20), Mal (18), KC (24), B (UK)	c.769C > T (E6)/ c.769C > T (E6)
P#18	**F 38**	AI (2), HPT (5), CMC (5), **8**	0	UD (5), EH (5), AL (5), KC (5), B(UK)	c.173C > A (E2)/ c.173C > A (E2)
Q#19	F 40	HPT (4), AI (10), CMC (childhood), **11**	HG (15), DT (33),	AG (29), AIH (36), V(UK), UD (UK), A (UK), DES	c.967_979del13 (E8)/c.967_979del13 (E8)
R#20	M 44	AI (11), HPT (11), CMC (child), **9**	0	EH, A, B (27), KC (33), DES (33), B (UK)	c.769C > T (E6)/c.769C > T (E6)
S#21	**F 44**	AI (10), CMC (17), **9**	HG (13), HP,	V (UK), UD (UK), AL (30), KC (44), AS (44)	c.967_979del13 (E8)/c.967_979del13 (E8)
T#22	F 45	HPT (4), AI (16), CMC (UK), **6**	HG (16),	AG, KC (45)	c.769C > T(E6)/c.977C > T (E8)
X#24	F 50	CMC (11), HPT (11), AI (11), **8**	HG,	Mal. (47), VI (11), EH (11), B (UK)	c.274C > T (E2)/c.967_979del13 (E8)
**≥ 7 manifestations**	**8 F/5 M** **Age range: 10-50 years** **Consanguinity:7/13**	**n = 13 patients ≥ 7 diseases**	**8/13 HG** **2 HT/13** **2 DT/13** **1HP/13**	**13/13**	8/26 : c.769C > T (E6)/ 5/26: c.967_979del13(E8) 4/26: c.958del (E8) 4/26: c.1193delC (E10) 2/6: c.173C > A (E2) 1/26: c.977C > T (E8) 1/26: c.274C > T (E2) 1/26: c.1616C > T (E14) **10 homozygous**

Patients are classified by family and increasing age (years) at baseline. The events are ranked in chronological order of appearance and followed by age of onset (years). The patients have been split in 2 groups according to the number of clinical manifestations < 7 (n = 12) or ≥7 manifestations (n = 13). Seven was the median number of clinical manifestations for the whole group. Patients with less of 7 clinical manifestations and those with 7 or more clinical manifestations were similar in age ranges: 8 to 52 and 10 to 50 years old, respectively. The number of female subjects seemed lower in the group with fewer than 7 manifestations (4 female/12 patients) as compared to the other group (8 female/13 patients), as well as the number of consanguineous family (1/12 vs 7/13). The number of homozygous patients was slightly lower in the group with fewer than 7 manifestations (7/12 patients) as compared to the other group (10/13 patients). If the frequency of the variant c.769C > T (E6) was nearly similar in the 2 groups (8/24 alleles and 8/26 alleles in the 2 groups), the frequency of the variant c.967_979del13(E8) was much higher in the group with fewer than 7 manifestations (10/24 alleles) than in the other group (5/26 alleles) whereas the frequency of the c.1193delC (E10) variant and of the c.958del (E8) were slightly higher in the group with a number of manifestations ≥7 (4/26 alleles for both, with 2/26 and 0/26 alleles, respectively, in the group with fewer than 7 manifestations). The distribution of rare variants (3 or 4 in each group) was similar in both groups. The number of endocrine and nonendocrine manifestations was much lower in the group (respectively 4/12 and 7/12 patients) with fewer than 7 disorders than in those with ≥7 (respectively 9/13 and 13/13).

Abbreviations: A, alopecia; AG, atrophic gastritis; AI, adrenal insufficiency; AIH, autoimmune hepatitis; AS, asplenia; B, bronchiolitis; CMC, chronic mucocutaneous candidiasis; DES, dry eye syndrome; DT, diabetes; E, Exon; EH, enamel hypoplasia; F, female; HG, hypogonadism; GD, Graves disease; HP, hypopituitarism; HPT, hypoparathyroidism; HT, hypothyroidism; I, intron; KC, keratoconjunctivitis; M, male; Mal, malabsorption; TIN, tubulointerstitial nephritis; UD, ungual dystrophy; UK, unknown; V, vitiligo.

As shown in Table 1, all variants were associated with the Whitaker triad (sometimes incomplete, probably in relationship with the age of diagnosis). Whatever the variant, all patients showed nonendocrine manifestations, emphasizing the importance of screening for them. Variants c.173C > A (E2), c.415C > T (E3), and c.977C > T (E8) of the *AIRE* gene were not associated with other endocrine manifestations. The *AIRE* variant c.653-70G > A (I5) was associated with isolated hypoparathyroidism but in a young patient.

Otherwise, Table 4 shows that patients with fewer than 7 clinical manifestations and those with 7 or more clinical manifestations were similar in age range: 8 to 52 and 10 to 50 years old, respectively. The number of female subjects seemed lower in the group with fewer than 7 manifestations (4 female/12 patients) as compared to the other group (8 female/13 patients), as well as the number of consanguineous family (1/12 vs 7/13). The number of homozygous patients was slightly lower in the group with fewer than 7 manifestations (7/12 patients) as compared to the other group (10/13 patients).

If the frequency of the variant c.769C > T (E6) was nearly similar in the 2 groups (8/24 alleles and 8/26 alleles, respectively), the frequency of the variant c.967_979del13(E8) was much higher in the group with fewer than 7 manifestations (10/24 alleles) than in the other group (5/26 alleles). The frequency of the c.1193delC (E10) and of the c.958del (E8) variants was slightly higher in the group with a number of manifestations ≥7 (4/26 alleles for both variants), in contrast to 2/26 and 0/26 alleles, respectively, in the group with fewer than 7 manifestations. The distribution of rare variants (3 or 4 in each group) was similar whatever the number of clinical manifestations. In addition, the number of other endocrine and nonendocrine manifestations was much lower in the group (respectively 4/12 and 7/12 patients) with fewer than 7 disorders than in those with ≥7 disorders (9/13 and 13/13, respectively).

To summarize, the patients with fewer than 7 clinical manifestations, despite being similar in age ranges to those with more than 7 manifestations, were more often male, belonging to nonconsanguineous families, with a slightly lower frequency of homozygous state and a 2-fold higher frequency of the variant c.967_979del13 (E8) than in the group with more than 7 manifestations, who more often presented other endocrine and nonendocrine diseases.

#### Follow-up

The median duration between inclusion and the last visit (V2-years) was 26 months (interquartile range 24-31). Two-year monitoring data were available for 22 of 25 patients. Our study lacks monitoring data for patients 5, 6, and 10 (V0 only). Three out of the 22 monitored patients could not be seen at 24 months but between 24 and 52 months. Their data were, however, considered as for V2-years visit.

Overall, at V1-year, 3 new manifestations (dry eye syndrome, oral mycosis, altered LFTs) occurred in 3 patients (#22, #19, #25, respectively). In addition, at V2-years, 5 new manifestations (hypergonadotropic hypogonadism, hypothyroidism, esophageal mycosis, enamel dystrophy, and splenic hypotrophy) had occurred in 5 patients (#7, #4, #25, #19, #23, respectively).

### Autoantibodies

#### Screening visit (V0)

The determination of some antibodies could not be performed in all patients for technical reasons (see detailed results in Table 5).

**Table 5.** Autoantibodies at inclusion visit

Manifestation	Autoantibodies	Autoantibody frequency in tested serums n/N (%)	Autoantibody frequency in tested patients with specific clinical manifestation n/N
Adrenal insufficiency	Anti-21 hydroxylase	14/21 (67)	13/22
Hypoparathyroidism	Anti-parathyroid	1/3 (33)	1/22
	Anti-calcium sensor receptor	2/19 (11)	2/22
	Anti-NALP5	7/18 (39)	5/22
Type 1 diabetes	Anti-GAD65	14/22 (64)	1/2
	Anti-ICA	5/14 (36)	0/2
	Anti-IA2	0/21	0/2
	Anti-insulin	3/20 (15)	1/2
Malabsorption	Anti-GAD65	14/22 (64)	6/8
Atrophic gastritis	Anti-gastric parietal cell	4/19 (21)	2/7
	Anti-intrinsic factor	7/19 (37)	4/7
Hypothyroidism	Anti-thyroglobulin	2/20 (10)	1/2
	Anti-thyroperoxidase	5/14 (36)	1/2
Testicular failure	Anti-testis	2/3 (66)	1/2
Ovarian failure	Anti-ovary	3/7 (43)	2/9
Autoimmune hepatitis	Anti-smooth muscle	1/19 (5.3)	0
Chronic mucocutaneous candidiasis	Anti-IL-22	15/18 (83.3)	15/22
	Anti-IL-17A	2/18 (11.1)	2/22
	Anti-IL-17F	13/18 (72.2)	13/22
Other autoantibodies	Anti-IFNλ	5/18 (27.7)	4/22
	Anti-IFNα	18/18 (100)	18/22
	Anti-IFNω	10/18 (55.6)	9/22

n/N: number of patients with the manifestation/total number of patients.

Abbreviations: anti-GAD65, glutamic acid decarboxylase 65-kilodalton isoform antibodies; anti-IA2, anti-tyrosine phosphatase antibodies; ICA, islet cell antibodies; IFN, interferon; IL, interleukin; NALP5, NACHT leucine-rich-repeat protein 5 antibodies.

Two of the 19 patients tested for anti-CaSR autoantibodies had detectable antibodies at baseline, and both had clinical hypoparathyroidism. One of the 3 tested patients for anti-parathyroid antibodies had detectable antibodies and presented with hypoparathyroidism. Of the 2 patients without anti-parathyroid antibodies, 1 did not have hypoparathyroidism. Seven of 18 patients tested were positive for anti-NALP5 antibodies, and 1 of the 2 remaining patients developed hypoparathyroidism during the 2-year follow-up.

For CMC, 15 of the 18 patients tested for IL-22 antibodies were positive, with all 15 having CMC. Two of the 18 patients tested for anti-IL-17A antibodies were positive, both of whom had CMC. Thirteen of the 18 patients tested for anti-IL-17F antibodies were positive, with all 13 patients showing CMC. All 18 patients tested for anti-interferon (IFN)-α antibodies were positive, and all had CMC. Ten of the 18 patients tested for anti-IFN-ω antibodies were positive, 9 of whom had CMC. Five of the 18 patients tested for anti-IFN-λ antibodies were positive, including 4 with CMC.

#### Correlation between phenotype and IL-22, IL-17A, and IFN-ω Autoantibodies

No clinical difference both in terms of number of other endocrine or nonendocrine manifestations or in terms of severity in mucocutaneous candidiasis were found between the patients with autoantibodies against IL-22, IL-17A, and IFN-ω (data not shown).

#### Follow-up

Regarding the monitoring of anti-islet autoantibodies, 1 of the patients who did not show any anti- glutamic acid decarboxylase (GAD), IA-2, or ICA autoantibodies at V0 became positive for anti-GAD at V1-year, and 2 patients became positive at V2-year, 1 for IA-2 and 1 for ICA. None of them developed type 1 diabetes, although 2 of 25 patients had type 1 diabetes at inclusion.

Two patients who did not have the other types of antibodies at V0 (anti-thyroid, gastric, adrenal, or liver) became positive at V1-year, 1 for anti-gastric parietal cell antibodies and 1 for anti-CaSR; 3 other patients became positive at V2-years, 1 for anti-thyroglobulin, 1 for anti-intrinsic factor antibodies, and 1 for anti-smooth muscle antibodies. None of these patients developed the corresponding clinical features.

### Immunophenotyping of Peripheral Blood Lymphocytes

Lymphocyte immunophenotyping was performed in 19 of 25 patients. The results are detailed in Tables 6, 7, and 8.

**Table 6.** Lymphocyte immunophenotyping compared with a control population*^a^*

Absolute number	APECED group	Control population*^a^*
	Median (IQR)	Normal range
Lymphocytes/mm^3^	2294 (1598-3446)	1500-4000
Lymphocytes CD3+/mm^3^	2014 (1310-2662)	1100-1700
Lymphocytes CD4+/mm^3^	1502 (767-1800)	700-1000
Lymphocytes CD8+/mm^3^	507 (373-912)	500-900
CD4/CD8 ratio	1.9 (1.5-2.6)	1-1.4
Lymphocytes CD56+/mm^3^	115 (76-185)	200-400

Abbreviations: APECED, autoimmune polyendocrinopathy-candidiasis-ectodermal dystrophy; IQR, interquartile range.

^*a*^The laboratory standards are those used routinely by the Lille University Hospital Immunology Laboratory at the Center for Biology Pathology.

**Table 7.** B lymphocyte immunophenotyping of patients younger and older than 30 years compared with a control group of healthy subjects of the same age

Lymphocytes	APECED< 30 years	Control< 30 years	*P*	APECED > 30 years	Control > 30 years	*P*
	Median (IQR)	Median (IQR)		Median (IQR)	Median (IQR)	
Total number of lymphocytes(10^3^/mm)	2466 (1492-3503)	1908 (1701-2298)	.61	2123 (1702-2856)	1825 (1567-2171)	.28
Percentage of B lymphocytes among the whole number of lymphocytes (%)	13.8 (5-19.9)	13.3 (9.8-18.2)	.67	3.5 (1.3-8)	11.7 (9.2-14.1)	**<**.**001**
B (10^3^/mm^3^)	353 (139-494)	261.2 (172-385)	.98	81.5 (17-157)	202 (152-299)	.**003**
Naive B (%)	61.7 (49.5-80.5)	75.3 (66.7-83.4)	.064	25.4 (7.9-48.4)	63.9 (52.8-72)	**<**.**001**
Naive B (10^3^/mm^3^)	69.9 (0-319.6)	201.9 (132-296)	.061	15.2 (0-39.6)	124 (96-190)	**<**.**001**
Nonswitched memory B (%)	5.3 (2.3-8.1)	3.1 (1.9-4.8)	.19	10.3 (3.7-20)	2.1 (1.5-3.3)	.88
Nonswitched memory B (10^3^/mm^3^)	6.9 (0-15.4)	8.2 (4.1-12.4)	.56	25.1 (0.7-14.6)	4.6 (2.5-8.7)	.26
Switched memory B (%)	8.1 (5.3-10.6)	4.5 (2-10.1)	.19	13.7 (5-17.1)	10.5 (7.9-17.3)	1
Switched memory B (10^3^/mm^3^)	13.3 (0-23.3)	13.7 (4.7-24.7)	.50	10.8 (2-23.6))	19.4 (12.6-36.1)	.058
Marginal zone B (%)	10.8 (5.7-18.1)	8.1 (5.6-14)	.23	21.6 (19.7-42.7)	14.8 (9-200.4)	.90
Marginal zone B (10^3^/mm^3^)	26.1 (0-38.1)	23.4 (14.9-36.1)	.66	7.2 (0-46)	26.6 (17.7-49.4)	.075

Abbreviations: APECED, autoimmune polyendocrinopathy-candidiasis-ectodermal dystrophy; IQR, interquartile range. Significative *P*-value are in bold.

**Table 8.** Number of patients and severity of the disease according to B immunophenotype

	APECED < 30 years old		APECED >30 years old	
Percentage of B lymphocytes among the whole number of lymphocytes (%)	**<9.8 :** **#13 (12)** **#15 (9)** **#5 (7)** #11 (5) #8 (3)	**>9.8 :** **#10 (10)** #9 (5) #6 (5) #2 (2) #1 (2) #3 (1)	**<9.2 :** **#19 (11)** **#21 (9)** **#20 (9)** **#24 (8)** #22 (6) #25 (6) #23 (4) #17 (5)	**>9.20**
B (10^3^/mm^3^)	**<172 :** **#13 (12),** **#15 (9)** #9 (5) #11 (5) #8 (3)	**>172 :** **#10 (10)** **#5 (7)** #2 (2) #1 (2) #6 (5) #3 (1)	**<152** **#19 (11)** **#21 (9)** **#24 (8)** #22 (6) #25 (6) #23 (4)	**>152** **#20 (9)** #17 (5)
Naive B (%)	**<66.7** **#13 (12)** **#15 (9)** #8 (3) #6 (5) #11 (5) #3 (1)	**>66.7 :** **#10 (10)** **#5 (7)** #2 (2) #1 (2)	**<52.8** **#19 (11)** **#21 (9)** **#20 (9)** **#24 (8)** #23 (4) #22 (6) #17 (5)	**>52.8** #25 (6)
Naive B (10^3^/mm^3^)	**<132** **#13 (12)** **#15 (9)** #9 (5) #11 (5) #8 (3) #3 (1)	**>132 :** **#10 (10)** **#5 (7)** #6 (5) #2 (2) #1 (2)	**<96** **#19 (11)** **#21 (9)** **#24 (8)** #22 (6) #17 (5) #25 (6) #23 (4)	**>96** **#20 (9)**
Nonswitched memory B (%)	**<1.9** #8 (3) #1 (2)	**>1.9** : **#13 (12)** **#10 (10)** **#15 (9)** **#5 (7)** #6 (5) #11 (5) #9 (5) #2 (2) #3 (1)	**<1.5** **#21 (9)** **#24 (8)**	**>1.5** **#19 (11)** **#20 (8)** #23 (4) #22 (6) #17 (5) #25 (6)
Nonswitched memory B (/mm^3^)	**<4.1** #8 (3)	**>4.1** **#13 (12)** **#10 (10)** **#15 (9)** **#5 (7)** #11 (5) #9 (5) #6 (5) #2 (2) #1 (2) #3 (1)	**<2.5** **#21 (9)** **#24 (8)** #25 (6)	**>2.5** **#19 (11)** **#20 (8)** #22 (6) #17 (5) #23 (4)

#X corresponds to each individual patient. Number between brackets represents the number of clinical manifestations; they are written in bold characters when the number of clinical manifestations is ≥ 7. Below 30 years old, there was no obvious differences in the number of patients and the number of clinical manifestations ≥7, according to the level of B lymphocytes (Percentage of B lymphocytes among the whole number of lymphocytes (%), B (10^3^/mm^3^), Naive B (%), Naive B (10^3^/mm^3^)), in contrast with patients over 30 years old, where both the number of the patients and the number of clinical manifestations ≥7 were higher in the B lymphopenic group.

Concerning “Nonswitched memory B (%)” and “Nonswitched memory B (/mm^3^),” the patients under 30 years old without deficit in “Nonswitched memory B” are more numerous and have a more severe clinical phenotype.

Abbreviations: Abbreviations: APECED, autoimmune polyendocrinopathy-candidiasis-ectodermal dystrophy.

#### Comparison of APECED patients and a control group

The lymphocyte immunophenotyping of the APECED group showed an increase of CD3+, CD4 +, and CD8+ T cell lymphocytes and NK lymphopenia compared with the normal range (Table 6).

The total number of B lymphocytes and the B-type subpopulation was similar between APECED patients < 30 years old and an age-matched control population. The B lymphocyte immunophenotyping of APECED patients >30 years old showed significant B lymphopenia, both in proportion (*P* < .001) and absolute numbers (*P* = .003) compared with an age-matched control population, associated with a naive B cell lymphopenia, both in proportion (*P* < .001) and in absolute numbers (*P* < .001). There was no significant difference for other B subpopulations (Table 7).

#### Correlation between phenotype and immunophenotyping

Then, to look for any relationship between immunophenotyping and clinical manifestations or disease severity, we used the lower limit of the interquartile range to compare the number of patients and the number of severe diseases (patients with ≥7 manifestations) according to B immunophenotype (Table 8).

Before 30 years old, there were no obvious differences in the number of patients and the severity of the disease according to

- Percentage of B lymphocytes among the whole number of lymphocytes (%)
- B (10^3^/mm^3^)
- Naive B (%)
- Naive B (10^3^/mm^3^)

In contrast, over 30 years old, the number of patients, and the number of patients with severe disease, was overtly higher in the group with low levels of

- Percentage of B lymphocytes among the whole number of lymphocytes (%)
- B (10^3^/mm^3^)
- Naive B (%)
- Naive B (10^3^/mm^3^)

Concerning nonswitched memory B (%) and nonswitched memory B (/mm^3^), the patients under 30 years old without deficit in nonswitched memory B were more numerous and had a more severe clinical phenotype.

## Discussion

To our knowledge, this is the first prospective study performed in APECED syndrome to systematically screen for all manifestations of the syndrome at each visit, including rare underdiagnosed and pauci-symptomatic manifestations.

We faced some difficulties with patient inclusion, due to the rarity of this little-known disease and to the challenge to convince both clinicians and patients to participate in a demanding prospective clinical study. The legal framework of this research study required patients to be investigated in an authorized, prespecified center, which was a major difficulty for a number of patients and led to underinclusion of both the most severe and the mildest forms of the disease. We thus elected to include patients past the 2-year follow-up to complete the study. This shows the need for adaptation of clinical research constraints for rare diseases.

Only slightly more than half of the 41 genetically confirmed cases (25 from 23 families) could be analyzed among a nationwide population of 64 million inhabitants. The first lesson of the flowchart is that 5 out of the 41 APECED patients died either before inclusion or after the end of the study; this corresponds to 8% of the cohort. Death occurred mostly in patients before the age of 40, underscoring the severity of the disease. Of the 5 deaths, 1 was from a probable acute adrenal insufficiency crisis when traveling abroad, 1 from respiratory insufficiency, and the other 3 from unknown causes. In addition, the most recent data of our cohort show a sudden death at 15 years old in a young lady who had well-balanced hypoparathyroidism and adrenal insufficiency and a severe oral malignancy in another patient. Of note, cases of sudden death, such as the one mentioned, are reported in several studies including patients doing well (25). Besides sudden hypocalcemia or adrenal crisis, autoimmune-induced myositis with atrioventricular block is an underinvestigated track that could deserve a more systematic screening, all the more so that it has also been reported under treatment with anti-programmed cell death-1 (26, 27). This high mortality rate is in accordance with 2 recent European reports (28, 29): 29/91 patients from the Finnish cohort died during the 47-year follow-up period, at a median age of 35 years, 10 from endocrine or metabolic diseases (diabetes excluded), 5 from oral and esophageal malignancy, 2 each from infectious and digestive diseases, 3 from circulatory or neurological diseases, 3 from alcohol-related diseases, and 4 from accidents. No suicide was observed (28). The mortality rate was 14.6% in the Italian cohort of 158 patients during a follow-up of 23 ± 15 years, at a mean age of 35 ± 21 years, one-third from cancer especially of the upper intestinal tract (29). So even if the follow-up of our cohort was much shorter than in the Finnish or the Italian cohort, we confirm the severe prognosis of the disease with a high mortality rate, assessed to be around 25% in more than a third of the studies reviewed in Garelli's publication (29).

Because of these limitations, only 25 APECED patients out of the 43 included were analyzed in the present work. The overall prevalence of the disease could be estimated at around 41/64 million inhabitants, ie, 1 case per 1.5 million inhabitants, which is very low even if probably underestimated.

Due to the usual autosomal recessive inheritance of the disease, a high rate of consanguinity was declared in our cohort [8/25 patients (32%), mainly from Africa] as in the Iranian Jewish cohort (12). The 17 cases of homozygosity suggest that the level of consanguinity might be higher, as also suggested by the higher frequency of APECED syndromes in isolated areas such as Finland (30) and islands such as Sicilia or Sardinia (29), despite an apparent low rate of consanguinity (5%) in this last study. Our study differs from other studies due to the multiple ethnic origins of the cohort, with about 70% of the patients of European descent and about 30% from the Maghreb region, West Africa, and the French West Indies. To date, there have been no studies on APECED syndrome in these areas of the world, except for India (25), but these results show that, besides Europe, the disease is also present in Africa.

Regarding the molecular profile of this cohort, 11 different *AIRE* variants were identified, whereas only 8 had been diagnosed in the Finnish cohort (30), 9 in the US cohort (18) of multiple historical ancestry, and more than 18 *AIRE* variants in the Italian cohort (29). The most common variant in our study is the “Finnish” variant *c.769C > T*, which represents 20% of alleles in the US cohort (18) and 11.8% in the Italian cohort (29). The *c.967_979del13* variant in exon 8 is the second most frequent in our study and the most frequent in the English, Norwegian, and Irish cohorts as well as in the northwestern French and US cohorts. However, it is not present in the Italian cohort, in good correlation with the presence of in mean fewer than 7 clinical manifestations in the Italian cohort (29). The genotype differences between these cohorts and the present study may be explained by the high proportion of Irish ancestors in the US cohort and the geographical and historical proximity of the northwest region of France with England and Ireland. Interestingly, APECED syndrome was present in other ethnic groups, as 3 consanguineous patients from Maghreb presented the *c.1193delC* variant (exon 10) in the homozygous state. This variant affects the proline-rich region and has been rarely described in the previously published cohorts. There is currently no descriptive study of patients with APECED syndrome in North Africa. The *c.1066del* variant (exon 9), found on 1 allele, has been described for the first time in our study in a patient from the French West Indies. This variant alters the zinc finger domain of the AIRE protein, which is the domain of transcription regulation, allowing chromatin decondensation. Patient #3, from a Roma community, also had a new variant: a deep intronic variant, *c.653-70G > A*, in intron 5, which has never been reported; it was found in the heterozygous state associated with the Finnish variant *c.769C > T*. This 3-year-old child had a different clinical presentation from the other cohort patients since she had only 1 clinical manifestation, isolated autoimmune hypoparathyroidism. She was included because she had been positively tested for the *AIRE* gene variant before inclusion. This phenotype has recently been reported in a family of heterozygous composite patients with an allele bearing the widespread variant *c.967_979del13* and an allele with intron insertion at a splice site of mRNA (*c.995 + (3_5)delGAGinsTAT*) (31). This same intronic insertion was described in an Italian series in the heterozygous state but was not associated with isolated hypoparathyroidism (32). Another mutation of intron 5 was recently reported in the homozygous state in a sibling from a consanguineous Spanish family (first cousin parents). They did not exhibit the isolated hypoparathyroidism phenotype (33). Given the young age of our patient, it is impossible to know if autoimmune hypoparathyroidism would be later associated with other APECED manifestations. To summarize, the variability of the *AIRE* gene molecular profile in our cohort appears greater than in the US cohort, with 2 new variants reported. This variability could be partly explained by the presence of immigrant patients of African origin coming from consanguineous families. Seventeen out of our 25 patients were homozygote, and 20 patients bore the widespread *c.769C > T* or *c.967_979del13* variants. A north-south and west-east gradient of the frequency of the disease was demonstrated, especially for the Finnish and Irish variants, probably related to the history of population migration. Interestingly, this knowledge can assist in prevention through genetic counseling, especially in populations where consanguineous marriage is frequent.

Regarding the clinical phenotype, the classic manifestations in our series (adrenal insufficiency, hypoparathyroidism, hypogonadism, and candidiasis) do not differ greatly from the other series [see (29) for review]. Regarding the diagnosis, ungual dystrophy and CMC are the 2 first manifestations but may be overlooked. The average number of manifestations of the syndrome is 7 in our study (ranging from 1 to 13 events) and 2 to 10 in the Perheentupa series (30) (although asplenia, nail dystrophy, and hypoplasia of tooth enamel had been removed from the count in this study). The median age at diagnosis is rather high (12 years old), as compared with previously reported studies, meaning that the diagnosis was in most cases done several years after the onset of the first symptoms (in mean at 7 years old). A high proportion of patients (76%) already presented the Whitaker's triad, which probably led to the diagnosis, especially before the systematic screening for the *AIRE* gene. This may be a limitation in the evaluation of the disease’s natural history but may also witness the severity of the disease, specifically when potentially severe nonendocrine manifestations were not screened for (malabsorption, pneumonitis, asplenia).

According to clinical reports, 2 patients (#4 and #17) had a history of trichophytosis caused by a parasitic fungus, which, unlike candidiasis, is not an opportunistic infection. As for the yeast *Candida albicans*, host immunity against *Trichophyton* relies in part on the Th17 immune response, which is altered in APECED (34). Fifty percent of patients in our study had antifungal therapy, at least intermittently, and neither mycological samples nor antifungal susceptibility testing results were available. Long-term antifungal treatments may favor the emergence of yeast strains resistant to azole antifungal agents but may also protect against cancers of the upper intestinal tract, since we observed a relatively low level of cancers in our study. This suggests the usefulness of the treatment and of the systematic study of yeast strains in this context. Specific biological markers would be useful such as yeast anti-glycan antibodies (against *Candida albicans* mannans, laminaribiose, *Saccharromyces cerevisiae*) (35) or soluble lectins (mannose-binding lectin, galectin-3, and pentraxin 3).

Nearly one-third (32%) of our patients had malabsorption, a frequency slightly higher than in most other series, probably reflecting our systematic screening. Also of note is that the term “intestinal dysfunction,” in part related to candidiasis infection, was used in the US cohort in which a higher incidence rate was found; this shows that the definition of malabsorption might be difficult in these patients. The causes were multiple and included celiac disease and exocrine pancreatic failure; the remaining causes were undetermined since no intestinal biopsy to test for lymphocytic infiltrates had been performed. Nevertheless, a previous study has shown a reduction of enteroendocrine cells, a specific and important early event in the pathogenesis of gastrointestinal dysfunction in APECED syndrome (36). In addition, the gut microbiota of patients with APECED is altered and enriched with predominantly gram-negative bacterial taxa that may promote biofilm formation and lead to increased exposure to lipopolysaccharides (a major component of gram-negative bacteria) in the patients (37). Malabsorption may favor undernutrition and frailty, especially in case of infection related to asplenia and immune alterations.

Asplenia was found in one-quarter of the patients, probably as a result of systematic screening (6 patients out of 23 screened), vs 19% in the Finnish series, 9% in the US series, and less than 5% in the Italian cohort, respectively (18, 29, 30). This relatively high frequency of asplenic patients should lead to preventive immunization against influenza, pneumococcus, meningococcus, and SARS-CoV-2, a point not addressed in previous studies (38).

Most of the patients (80%) had ectodermal dystrophy, with a sex ratio (male/female) of 1.4. This is one of the most frequent and early manifestations occurring in childhood, often before the classical triad. Ectodermal dystrophy is not often seen in the literature series and is probably underdiagnosed. In our study, 28% of patients had vitiligo, which is in accordance with most reports [31% in the Finnish cohort (30), 37% in the US cohort (18] but not all [only 17% in the Italian cohort (29) and 9% in the Russian cohort (39]. Fifty-two percent had nail dystrophy, as compared with only 17% in the US series (18) and 25% in the Italian study (29), but the diagnosis may be difficult to differentiate from onychomycosis. Enamel hypoplasia was found in 48%, a lower frequency than in the Finnish series (77%) (30), however higher than in the Italian study (around 23%) (29). Keratoconjunctivitis was found in 33%, including 3 of the 8 patients with dry eye syndrome, as compared to 25% in the Italian study (29). No cases of cataract or iridocyclitis were identified. Ocular involvement should be systematically searched, given its few symptoms and the risk of slow progression to blindness.

LFTs could only be performed in half of the patients at inclusion, and 62% of LFTs were abnormal, a frequency higher than reported in the literature (40% or less) (18). Half of the patients with LFT abnormalities were symptomatic, without histological documentation. This might be due to the older age of our cohort (15 adults/25 subjects). In the literature, bronchial biopsies have shown pulmonary lymphocyte infiltration damaging the alveolocapillary membrane, which could explain the decreased diffusing capacity of the lungs for carbon monoxide in these patients (40). Pulmonary involvement is potentially serious since 1 of the excluded patients—who was a smoker—died of severe pulmonary fibrosis, as reported in other cases (41). Early detection of pulmonary complications, both clinically and with LFTs or low-dose chest computed tomography, would probably be useful in further follow-up, supporting smoking cessation, etiological investigation, and pulmonary rehabilitation. The use of immunomodulators such as azathioprine and anti-CD20 (Rituximab) has been reported in some cases of lung fibrosis in APECED patients (41, 42), but this is not the classic treatment of pulmonary fibrosis (43). Indeed, the mechanism of lung involvement in APECED is an autoimmune pneumonitis (43, 44), often associated with opportunistic infection such as pneumocystis or aspergillosis. The potential similarities with anti-programmed cell death 1 inhibitor-related pneumonitis have not been studied to our knowledge (45).

Other rarer manifestations of APECED syndrome were identified, including tubulointerstitial nephritis (patient #4) and Devic's disease (patient #21). This demyelinating autoimmune inflammatory disease of the central nervous system is characterized by the presence of myelin oligodendrocyte glycoprotein autoantibodies and anti-aquaporin-4 antibodies, which have a direct pathogenic role. The disease has never been described in the literature associated with APECED syndrome, although other central nervous system disorders, such as Vogt-Koyanagi-Harada disease, have been reported. At the time of this study, none of our patients had been found to have autoimmune generalized lipoatrophy (46).

To summarize phenotypic findings, besides the classical Whitaker's triad, APECED should be evoked as the etiological diagnosis of any isolated case of hypoparathyroidism. Malabsorption, asplenia, and pulmonary manifestations should be systematically searched since they can favor malnutrition and frailty and play a part in the high mortality rate. The numerous manifestations of the disease seem to have important psychosocial consequences, making follow-up difficult. Indeed, depressive symptoms affect 29% of patients, and general health, emotional well-being, and vitality were the most diminished aspects of quality of life in a previous work in adult Finnish patients, in correlation with age and duration of the disease (47). The implementation of a quality-of-life questionnaire and psychological support would help to take into account the consequences of the disease.

Biological phenotyping of this APECED cohort included both a large antibody panel and lymphocyte immunophenotyping. The antibody panel can help orient toward an autoimmune etiology and therefore toward APECED syndrome at the early stage of the disease, even if the specificity and sensitivity of some antibodies are not as good as previously reported. Nevertheless, it is not clear whether early detection can be useful in preventing complications.

There was no difference in the prevalence of classical antibodies compared with the other series. The measurement of anti-CaSR antibodies seemed not very sensitive for the diagnosis of hypoparathyroidism in our study, as these autoantibodies were present in only 2/19 patients with hypoparathyroidism. The anti-NALP5 antibodies, described in the literature as being very specific for APECED syndrome (100%) and a good predictive marker of parathyroid involvement (48-51), were present in only 7/18 (39%) patients tested and in 5/22 (23%) patients with hypoparathyroidism. One patient without hypoparathyroidism had detectable levels of antibodies and did not develop hypoparathyroidism in the follow-up period, which might reflect subclinical autoimmunity. Similarly, the high prevalence of anti-GAD antibodies (64% positive) was discordant with the low prevalence of type 1 diabetes (only 2 patients), as underlined in Garelli's study (29).

Most patients (15/18, 83%) were positive for anti-IL-22 antibodies (all positive for CMC), and most (13/18, 72%) were also positive for anti-IL-17F antibodies (all with CMC). Only 2/18 (11%) CMC patients were positive for anti-IL-17A antibodies and were also positive for the other antibodies (IL-22 and IFN-ω). These results are consistent with those of Kisand et al on ∼162 patients (52). Autoantibodies against IL-22 and IL-17F seem to be correlated with chronic mucocutaneous candidiasis as previously reported (52), suggesting that Th17 cytokines are central in human epithelial immunity against candida infection.

Anti-IFN-ω antibodies were present only in a little more than 50% of our patients, at difference with the first extensive study on the relevance of anti-IFNs antibodies in the APECED patients (53), where anti-IFN-ω antibodies were present in almost all patients and with higher titers as compared to IFN-alpha antibodies. This discrepancy may be due to methodological differences but also to different phases of the disease evolution (with variations of anti-IFNs antibody titers with time). Therefore, anti-IFN-ω antibody assay does not seem to be the most specific diagnosis test for APECED, and *AIRE* molecular study appears definitely as the main diagnosis criteria.

No obvious clinical difference both in terms of number of other endocrine or nonendocrine manifestations or in terms of severity in mucocutaneous candidiasis was detected between the patients according to the presence or absence of autoantibodies against IL-22, IL-17A, or IFN-ω. IL-22 antibodies seem to be the most specific for the diagnosis.

Also, our study was carried out before the COVID-19 pandemic, but since then we have seen the importance of these anti-interferon antibodies in the occurrence of pneumopathy. For example, autoantibodies neutralizing type I IFNs present before SARS-CoV-2 infection confer a very high risk of critical COVID-19 (54). These observations suggest that immunomodulatory treatment such as intravenous immunoglobulins or anti-CD20 (Rituximab) could reduce susceptibility to infections (eg, candidiasis, severity of viral infections). Nevertheless, this hypothesis is not concordant on the one hand with the fact that APECED patients are already B lymphopenic, while, on the other hand, many APECED patients harbor extremely high-affinity, neutralizing autoantibodies, particularly against specific cytokines such as type I IFNs that showed a striking inverse correlation with type I diabetes, suggesting that naturally occurring human autoantibodies may actively limit disease and be of therapeutic utility (55).

We found B and NK cell lymphopenia with a significant increase of CD4 + and CD8+ T lymphocytes, and the B cell responses have been shown downregulated in APS-1 patients (56). These results confirm in a larger cohort, those found in an Italian study of 25 APECED patients (57), and those found in a US APECED cohort older than 15 years but not in cases with a shorter course (18). These findings are in accordance with our results in APECED patients older than 30 years. In the present series, this lymphopenia was found in the naive B lymphocyte population, both in proportion and in absolute counts, and not in other populations. We thus observed an age-dependent alteration of lymphocyte homeostasis. Naive B lymphocytes, endowed with immunoregulatory properties (58), are therefore significantly decreased, unlike memory B cells. This inversion of the naive B cell/memory B cell ratio has already been reported in the oldest APECED patients (57) and is similar to that found in systemic lupus erythematosus (59), suggesting a nonspecific immune mechanism (60). B and naïve B lymphopenia was clearly associated with a higher number of APECED cases with a higher number of clinical manifestations above 30 years old (but not below 30). In contrast, switched B lymphopenia was associated with a lower number of APECED cases with a smaller number of clinical manifestations below 30 years old (but not above 30).

## Conclusion

The results of this national, prospective, observational study confirm that autoimmune polyendocrine endocrine syndrome type 1, or APECED syndrome, is not only a very rare but also a severe disease with a high mortality rate, justifying close follow-up. The phenotypic variability is high, and the multiplicity of the manifestations suggests the need for multidisciplinary lifelong follow-up. This follow-up needs to be better codified, including systematic baseline LFTs or low-dose chest computed tomography, search for opportunistic lung infection if symptoms, abdominal ultrasound to test for asplenia, ophthalmologic examination, and intestinal investigation in case of diarrhea. The disease can be revealed by isolated hypoparathyroidism. The frequency of disease components such as bronchiolitis obliterans, keratoconjunctivitis, and splenic atrophy is underestimated because they are not tested for, as shown in this prospective study. This French cohort also shows large genotypic variability, with 11 different *AIRE* variants, including 2 new variants, related to the different ethnic origins, especially African, of this cohort. Homozygosity was present in 17/25 patients suggesting a higher level of consanguinity than that one previously reported (8/25 patients). Phenotype-genotype correlations show that patients with fewer than 7 clinical manifestations, despite being similar in age ranges to those with more than 7 manifestations, were more often male, belonging to nonconsanguineous families, with a slightly lower frequency of homozygous state and a 2-fold higher frequency of the variant c.967_979del13(E8) than in the group with more than 7 manifestations. An antibody panel can help orient toward an autoimmune etiology and therefore toward APECED syndrome at the early stage of the disease, even if the specificity and sensitivity of some antibodies were not as good as previously reported, with IL-22 seeming the most frequently identified in CMC. An age-dependent alteration of B lymphocyte homeostasis with B lymphopenia and an inversed naive B cell/memory B cell ratio was found, and further investigations are needed to confirm the role of B lymphocytes and to discover new therapeutic targets such as anti-CD45RC antibody (6) or pharmacologic inhibition of IFN-γ or Janus kinase-STAT signaling (61) to ameliorate mucosal fungal disease in APECED syndrome. Indeed, the treatment of 3 APECED patients with a Janus kinase inhibitor (ruxolitinib) has recently shown very interesting results in a pilot study (62). Close clinical and biological monitoring of APECED patients could be a useful strategy in forming an earlier diagnosis and preventing infections through immunization, favoring earlier treatment of pulmonary involvement and malabsorption and specifically targeted therapeutic pathways.

## Data Availability

The data that support the findings of this study are available on request from the corresponding author (L.H.). The data are not publicly available due to their containing information that could compromise the privacy of research participants.

## Clinical Trial Information

EudraCT registration no. NCT03751683.
